# Leveraging genomic redundancy to improve inference and alignment of orthologous proteins

**DOI:** 10.1093/g3journal/jkad222

**Published:** 2023-09-28

**Authors:** Marc Singleton, Michael Eisen

**Affiliations:** Howard Hughes Medical Institute, University of California Berkeley, Berkeley, CA 94720, USA; Howard Hughes Medical Institute, University of California Berkeley, Berkeley, CA 94720, USA; Department of Molecular and Cell Biology, University of California Berkeley, Berkeley, CA 94720, USA

**Keywords:** orthology, multiple sequence alignment, HMM, Drosophila

## Abstract

Identifying protein sequences with common ancestry is a core task in bioinformatics and evolutionary biology. However, methods for inferring and aligning such sequences in annotated genomes have not kept pace with the increasing scale and complexity of the available data. Thus, in this work, we implemented several improvements to the traditional methodology that more fully leverage the redundancy of closely related genomes and the organization of their annotations. Two highlights include the application of the more flexible *k*-clique percolation algorithm for identifying clusters of orthologous proteins and the development of a novel technique for removing poorly supported regions of alignments with a phylogenetic hidden Markov model (phylo-HMM). In making the latter, we wrote a fully documented Python package Homomorph that implements standard HMM algorithms and created a set of tutorials to promote its use by a wide audience. We applied the resulting pipeline to a set of 33 annotated *Drosophila* genomes, generating 22,813 orthologous groups and 8,566 high-quality alignments.

## Teaser

Though the identification of orthologous proteins is a key step in many comparative genomics analyses, methods for inferring and aligning their sequences in annotated genomes have not kept pace with the increasing scale of the available data. Thus, Singleton and Eisen implemented several improvements to the traditional methodology that more fully leverage the redundancy of closely related genomes and the organization of their annotations. The authors applied the resulting pipeline to 33 annotated Drosophila genomes, generating 22,813 orthologous groups and 8,566 high-quality alignments.

## Introduction

Comparative genomics is a powerful tool for yielding insights into evolutionary relationships, molecular function, and the forces that drive gene, genome, and population evolution. These methods often rely on the identification of homologous sequences or homologs, that is sequences with common ancestry, since this ensures that differences between sequences reflect variations in evolution from a common point of divergence. However, many analyses impose the additional condition that the sequences have diverged through speciation events (orthology) rather than duplications (paralogy) or other mechanisms such as horizontal gene transfer (Fitch 1970). The underlying assumption is that orthologs have conserved equivalent functions whereas paralogs, by virtue of their redundancy, are more likely to diverge (Ohno 1970; Nowak et al. 1997; Altenhoff et al. 2012; Pegueroles et al. 2013; Soria et al. 2014). This relationship between orthology and function is an essential component of modern biological research since it permits the transfer of annotations between biological systems using sequence similarity alone.

Given this importance, methods for inferring orthologous groups of proteins were developed shortly after the first genomes were sequenced in the late 1990s (Fleischmann et al. 1995; Goffeau et al. 1996; Consortium 1998). One early and influential approach was to cluster triangles of hits resulting from homology searches between pairs of genomes (Tatusov et al. 1997). Graph-based approaches have remained popular, and in the intervening years, many other researchers have refined this method by implementing various pre and postprocessing steps. Despite these improvements, many databases and pipelines use the same triangle clustering algorithm (Jensen et al. 2007) or other methods which require relatively few hits between sequences to infer an orthologous group, e.g. connected components and other single-linkage criteria (Remm et al. 2001; Linard et al. 2011; Train et al. 2017; Cosentino and Iwasaki 2018) or Markov clustering (Enright 2002; Li et al. 2003; Emms and Kelly 2015). However, the scale of biological sequence data has changed dramatically. For example, in the last decade, the number of annotated genomes available from NCBI has increased nearly 20-fold and currently exceeds 900 (O'Leary et al. 2016, Supplementary Fig. 1). Though this figure is only a rough proxy of the total number of assemblies available, it will likely continue to grow rapidly in the coming years as many large-scale genome assembly efforts such as i5K (Thomas et al. 2020), the Bird 10,000 Genomes Project (Feng et al. 2020), and the Vertebrate Genomes Project (Rhie et al. 2021) have already yielded results. Thus, the dense taxonomic sampling made possible by these projects poses new challenges and opportunities for the standard methods of orthology inference and alignment, which implicitly assume fewer and more distantly related genomes or fail to fully leverage the redundancy and organization of their annotations.

In this work, we therefore developed a computational pipeline that can robustly infer and align orthologous groups of proteins even when the genomes are highly redundant. Like many other orthology inference pipelines, our overall approach is based on clustering a graph of hits from homology searches. However, we modified many details to maximize the detection of highly diverged orthologs while also minimizing the impact of incomplete or incorrect annotations. Furthermore, since modern genome annotation pipelines frequently produce gene models and protein sequences in tandem, we implemented an additional clustering step to organize the resulting orthologous groups of proteins into gene-level units. However, most of our efforts were focused on the final step of aligning the orthologous sequences. Though genome annotation pipelines are often proficient at identifying the overall locus of genes, the accurate identification of exon boundaries and start codons when transcript evidence is limited remains an ongoing challenge (Frankish et al. 2015; Dunne and Kelly 2018). Consequently, protein sequences derived from annotation pipelines can include nonhomologous segments of significant length or exclude highly conserved segments. Such heterogeneity in the structure and length of the sequences in an orthologous group poses many challenges for their alignment and subsequent analysis. Thus, we implemented several novel quality control and data cleaning steps to correct mis-alignments and identify likely sequencing, assembly, or annotation errors.

To develop these methods, we chose a set of 33 assembled and annotated *Drosophila* genomes, which includes all 12 species from the original *Drosophila* 12 Genomes Consortium (Consortium 2007). However, the genomes of these 12 species have been re-sequenced since their first release, which has resulted in substantial improvements in their assemblies and annotations (Miller et al. 2018; Yang et al. 2018). Despite these developments and other several other recent genome assembly projects of species in the *Drosophila* genus, there is not yet a collection of high-quality alignments of orthologous proteins that reflects these improvements in genome assembly and diversity (Kim et al. 2021). Given the *Drosophila* genus spans diverse habitats and over 50 million years of evolution but maintains a conserved life cycle and body plan, such a resource would facilitate a new generation of studies that illuminate the forces that drive protein evolution in unprecedented detail (Wiegmann et al. 2011; Obbard et al. 2012).

## Materials and methods

### Sequence de-duplication and BLAST search parameters

The protein sequences for each annotation were de-duplicated by removing any sequences which had already appeared in association with the same gene. Thus, the first accession associated with a sequence and gene pair was the sequence's representative accession for the gene. BLAST+ 2.13.0 was used for the sequence similarity searches (Camacho et al. 2009). An E-value cutoff of 1 was used for the initial searches. However, this cutoff was lowered to 1E−^10^ during processing of the BLAST output.

### Extraction of HSPs from BLAST output

To reduce the computational burden of merging high-scoring segment pairs (HSPs) into hits, the BLAST output was filtered to extract HSPs associated with the highest-scoring gene. The HSPs were first grouped by target protein, and the resulting groups were sorted in descending order by the bit score of their highest-scoring HSP. Iterating over the groups, all HSPs in a group were passed to the next step until the parent gene of the group was not the parent gene of the highest ranked group. This method collected all candidate HSPs for a target gene if the highest-scoring HSP within a group exceeded the highest-scoring HSP of the next best gene. This is in some senses an extension of the best hit criterion where hits are considered at the level of genes rather than proteins. If multiple genes tied for the highest-scoring HSP, the iteration stopped when the parent gene of the current group matched none of these highest-scoring genes.

### Merging of HSPs into hits

Because BLAST can return multiple potentially overlapping HSPs for a query and target sequence pair, HSPs were merged into a single “hit” object that encompassed information from HSPs without overlap (“disjoint”) and HSPs with an acceptable amount of overlap (“compatible”). Hits were created by iterating over the HSPs in 2 passes. In the first pass, proceeding from highest to lowest bit score, HSPs were marked as “disjoint” if they did not overlap with any other HSP previously marked as disjoint. Although choosing the highest-scoring subset of HSPs is a kind of “scheduling problem” with a known solution, we implemented a simple greedy strategy to prevent fragmentation by prioritizing fewer long and high-scoring HSPs over many short and low-scoring HSPs. In the second pass, all HSPs in the disjoint set were first marked as “compatible,” and proceeding from highest to lowest bit score, the remaining HSPs were marked as compatible if the overlap with any other compatible HSP was no more than 50% of the length of either.

The best hit for each query was chosen as the hit with the highest sum of bit scores from only disjoint HSPs. The best hits were filtered by overlap and reciprocity criteria. The overlap criterion was applied first and required that 50% of residues in the query were aligned in compatible HSPs. This excluded false positives from conserved domains embedded in larger nonhomologous proteins by ensuring that the hits spanned a sufficient fraction of the query and target sequences. The reciprocity criterion required each query–target pair had a corresponding hit where the roles were reversed, which ensured that there was no ambiguity in which target was the best match for the query.

### Clustering by *k*-clique percolation

*k*-Clique percolation was implemented in 2 steps. In the first, maximal cliques were identified. In the second, a percolation graph was constructed by defining edges between cliques if they had *k* − 1 nodes in common. Clusters were the connected components of this second graph. The first step used the NetworkX implementation of a maximal clique algorithm. The second step used a modification of the NetworkX implementation of the *k*-clique community algorithm. The NetworkX implementation exhaustively finds all edges in the percolation graph. Since joining a *k*-clique community only requires that a clique has a single edge connecting it to that a community, this approach was needlessly expensive for large graphs. The custom implementation instead used a progressive approach where each clique was checked against a list of known communities, merging communities as necessary in each step.

The hit graph was sparse, so these algorithms were efficient when applied to its individual connected components. However, some components had a structure with many maximal cliques, which dramatically slowed the first or second step of the clique percolation algorithm. Thus, if either step exceeded 90 s, the process timed out, and the simpler *k*-core algorithm was used instead. Out of over 10,000 connected components, only 7 timed out, and many of those contained highly dense clusters of histone sequences.

### Addition of paralogs to orthologous groups

The protein sequences for each annotation were searched against themselves with the same settings as for the inter-genome searches. The resulting output was processed identically except the HSPs were not filtered using the best gene criterion. Thus, all HSPs for each query were merged into hits. The best hit for each query and target gene was chosen as the hit with the highest sum of bit scores from disjoint HSPs (grouping by target gene ensured that only the highest-scoring isoform was selected). Query–target pairs whose hits exceeded the maximum bit score for all inter-genome hits associated with that query and passed the overlap and reciprocity filters were designated as paralogous pairs. The orthologous groups were supplemented with paralogs by adding the paired sequences for each of the original members of the orthologous group.

### Initial alignment and selection of representative sequences

The sequences in each orthologous group were aligned using MAFFT 7.490 with the following settings: –globalpair –maxiterate 1000 –thread 1 –anysymbol –allowshift –leavegappyregion –unalignlevel 0.4 (Katoh and Standley 2013). Representative sequences for each gene were selected by maximum likelihood according to binary profiles constructed from these alignments. First each sequence was coded into gap and non-gap symbols. The sequences were grouped by gene, and for each group and position if at least 1 sequence was aligned in the group, the group contributed 1 count for the non-gap symbol to the profile at that position. Otherwise, the group contributed a count for the gap symbol at that position. To account for the phylogenetic dependencies between sequences, the counts were weighted according to a Gaussian process over the GTR2 consensus tree described in the section on inferring species trees (Altschul et al. 1989). If a species had multiple genes in the orthologous group, the species weight was divided evenly among them. Each coded sequence was scored according to this profile, and the maximum likelihood sequence for each gene was selected as its representative. By assigning a non-gap count to groups and positions where at least 1 sequence was aligned, the profile prioritized the selection of sequences with the fewest gaps that best matched the consensus alignment. Since this scheme can cause a sequence to score negative infinity if it has a gap at a position where every group has at least 1 aligned sequence, the profile was initialized with a pseudocount of 0.005 for the gap and non-gap symbols at each position.

### Alignment refinement

The representative sequences in the single copy orthologous groups were aligned with the same settings as described in the previous section except *a*_max_ was set to 0.7. A binary profile was created from the alignment using Gaussian process sequence weighting as described in the section on selecting representative sequences (because the orthologous groups were single copy and contained only representative sequences, each sequence received the full weight associated with its species). The binary profile was converted into a binary mask by identifying where the weighted fraction of non-gap symbols exceeded 0.5. The binary mask was closed with a structuring element of size 3, and highly conserved regions were identified as the contiguous intervals of this closed mask with a minimum length of 10. Diverged regions were taken as the complement of the highly conserved regions. For each diverged region, the corresponding segments of the sequences in the initial alignment were extracted and aligned with *a*_max_ set to 0.4. The resulting sub-alignments were stitched into the initial alignment.

### Alignment curation

The alignments were coded into gap and non-gap symbols to simplify the emission distributions. The “insertion” phylogenetic hidden Markov model (phylo-HMM) was composed of the 4 hidden states described in the main text. The emission distributions for each consisted of 2 components which modeled the gap pattern and the propensity for those patterns to remain constant (“gap stickiness”), respectively. The first component was a 2-state Markov process which was in turn composed of 2 subprocesses. The first was a phylogenetic process on the on the GTR2 consensus tree described in the section on inferring species trees, and the second was jump process at the tips. The second component was a beta-Bernoulli distribution on the number of symbols which were constant between subsequent columns. The “missing data” phylo-HMM was composed of 2 hidden states which were both parameterized with the same 2-state, 2 component Markov process as the insertion phylo-HMM. However, the emission probabilities were calculated as the posterior probability of the observed symbol given the data rather than the probability of the data. Only the alignments of the single copy orthologous groups were curated, so each tip in the species tree corresponded to a single sequence in the alignment.

The likelihoods of phylogenetic trees were efficiently calculated with Felsenstein's pruning algorithm, and all HMM algorithms were implemented with custom code which is available as the package Homomorph on PyPI (Felsenstein 1981). The insertion phylo-HMM was trained on 53,518 manually labeled columns in 15 alignments, and the missing data phylo-HMM was trained on 67,360 manually labeled positions in 23 sequences in 11 unique alignments (Supplementary Figs. 4 and 6). Because maximum-likelihood estimation of the model parameters yielded posterior decoding curves which toggled between hidden states too rapidly, the models were instead trained discriminatively (Krogh and Riis 1999). The difference, briefly, is maximum-likelihood estimation finds the parameters that best reproduce the observed distributions whereas discriminative training finds the parameters that minimize prediction error. Discriminatively trained models typically perform better in practice since real-world data are rarely fully described by the distribution specified by the model.

The posterior distributions over states were calculated for each alignment using the trained insertion phylo-HMM. Regions with a high probability of state 2 or 3 were candidates for trimming. However, because the probability of a state can change rapidly or gradually depending on the local context, a simple cutoff would not necessarily define the boundaries of these regions as the columns where the gap pattern changed most abruptly. Instead, the following algorithm was used. First, a high cutoff defined a “seed” region. The left and right endpoints of the seed were then expanded both inwards and outwards to define 2 intervals from which boundaries were selected. The outward expansion halted when the probability or its derivative was below 2 distinct thresholds, respectively. The inward expansion halted when the derivative was below a different threshold. The left and right boundaries were chosen as the columns in each interval with the maximum product between the derivative and the change in the gap profile between columns. The gap profile was calculated as the number of gaps in each column using Gaussian process sequence weighting as described in the section on selecting representative sequences. By combining where the model's confidence changed rapidly with the observed change in the gap pattern, this method generally selected reasonable boundaries.

States 2 and 3 have distinct characteristics which required different trimming strategies. Regions with a high probability of state 3 were handled first. Because the long poorly supported segments in state 3 regions were sometimes aligned to highly conserved columns or short segments in other sequences, trimming columns entirely would remove these segments from the other sequences even if they would not qualify as long and poorly supported themselves. Thus, regions with high state 3 probabilities were trimmed at the level of individual sequences rather than entire columns using the following method. First, the probability of state 3 for columns with a gap profile value less than or equal to 0.1 was set to 0 to break long poorly supported segments aligned to highly conserved columns into separate regions. Regions were defined with the previously described algorithm using high and low cutoffs of 0.75 and $1\times 10^{-3}$, respectively. The outer and inner derivative cutoffs were both 0.001. The mean number of non-gap symbols in a region was calculated using Gaussian process sequence weighting as described in the section on selecting representative sequences (the sequences with the 5 most non-gap symbols were also excluded to not bias the estimate with long poorly supported segments). The final mean *μ* was taken as the minimum of this value and 2. A cutoff *k*, derived from a geometric model of the number of non-gap symbols and a significance level *α*, was calculated using the equation k=log(α)/log(1−p)−1 where $p=\frac{1}{\mu +1}$ and α=0.01. Any sequence whose number of non-gap symbols in the region equaled or exceeded this value was trimmed by replacing all non-gap symbols with gaps.

To trim the remaining state 2 regions, the posterior probability of state 2 was added to a modified state 3 probability which was set to zero for any state 3 regions identified in the previous step. This ensured that any regions which were intermediate between states 2 and 3 were included. Regions were defined from this combined probability using the algorithm described previously except with a high probability cutoff of 0.9 instead of 0.75. The posterior probabilities from the missing data phylo-HMM were converted into state assignments using a similar method. However, the initial seeds were defined with a cutoff of 0.75, and the seeds were expanded outward to the first non-gap symbol or until the posterior probability of the “missing data” state was below 0.05. These assignments, which are available in the Supplementary Data, can be used to filter segments or entire sequences from downstream analyses.

### Inference of species trees

The orthology inference pipeline was first repeated with the outgroup species *Scaptodrosophila lebanonensis*. Orthologous groups with 1 sequence for each species were aligned, and 100 meta-alignments were constructed by randomly sampling 10,000 columns from these 9,435 alignments (the alignments were not refined before sampling). To determine the effect of invariant columns and gaps, 2 sampling strategies were used where invariant columns were allowed or disallowed and the maximum fraction of gaps was set at 0, 50, and 100%. Their combination yielded 6 different sets of meta-alignments. A tree was fit to each meta-alignment with the LG substitution model (Le and Gascuel 2008), 4 discrete gamma rate categories (Yang 1994), and optimized state frequencies using IQ-TREE 1.6.12 (Nguyen et al. 2014). If the sampling strategy allowed invariant columns, an invariant rate category was included. The resulting trees from each set were merged into a majority consensus tree (Supplementary Fig. 8). All figures are derived from the maximum 50% gap fraction meta-alignment set unless otherwise noted.

To fit trees using the GTR model of nucleotide substitution, the protein alignments were converted to nucleotide alignments using the corresponding coding sequences in the genome annotations. Some protein sequences were “low quality,” meaning that their coding sequences contained frameshifts, premature stop codons, or other errors even though they were strong hits to known protein-coding genes. The NCBI annotation pipeline corrects some of these defects in the protein sequences, which can complicate a simple “reverse translation” of the alignment. After rejecting alignments where the expected translation from a coding sequence differed from its corresponding protein sequence, 3,425 alignments remained. Consensus tree were derived from meta-alignments sampled from these alignments using the approach described previously except the GTR model was used in place of the LG model.

To fit trees using the 2-state GTR model of substitution, the protein alignments were first coded into gap or non-gap symbols. As before, 100 meta-alignments were constructed from these coded alignments for each sampling strategy. In this case, only the presence of invariant columns was varied, yielding 2 sets of meta-alignments. Trees were fit using the GTR2 model with no rate categories. An invariant category, however, was included if invariant columns were allowed. Since the bootstrap confidences were sometimes lower than 50%, the resulting trees from each set were merged into a loose consensus tree to prevent multifurcations (Supplementary Fig. 9).

## Results

### Pipeline overview

**Fig. 1.**
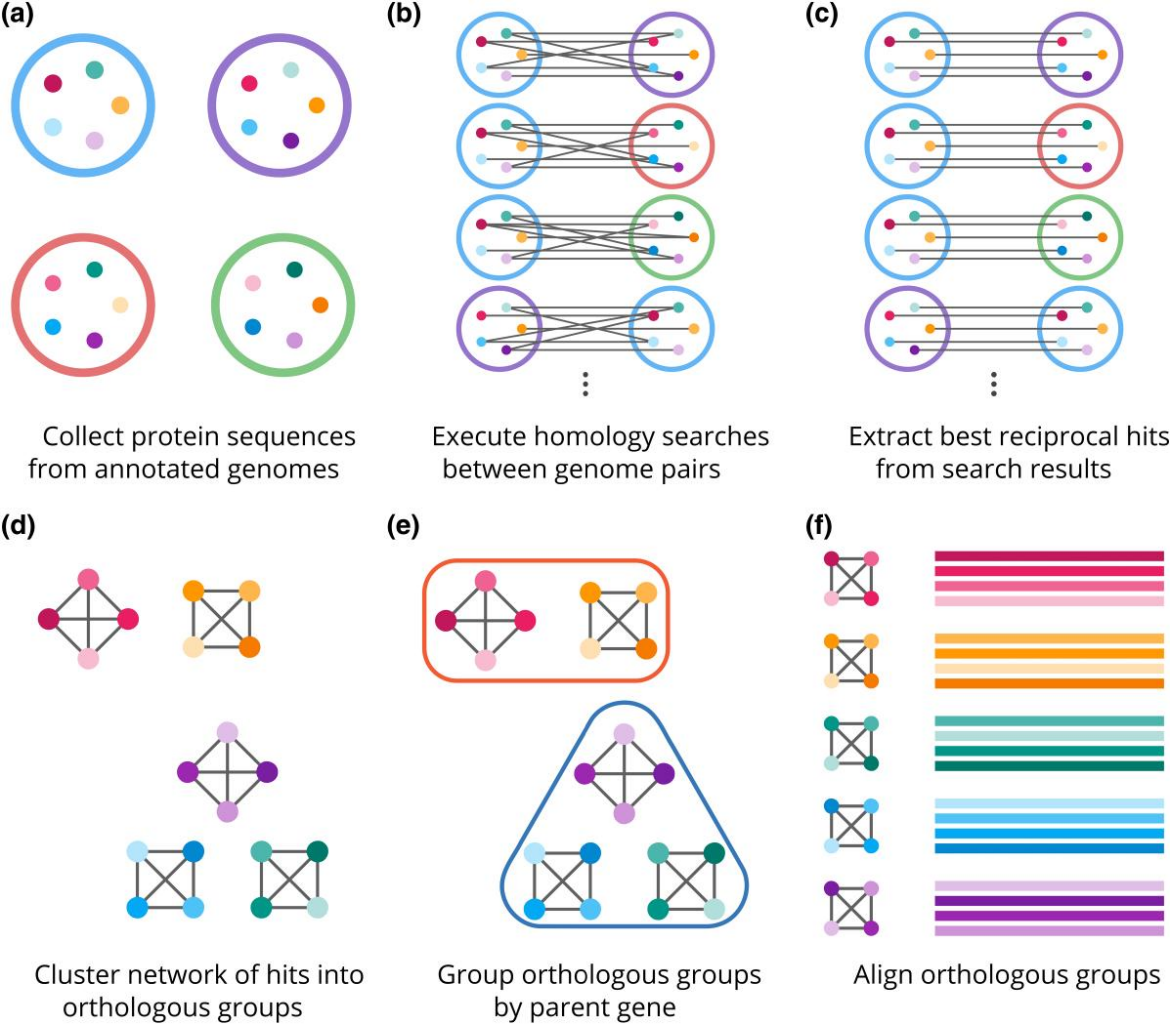
Overview of orthology inference pipeline. a–c) The large, open circles represent the annotated genomes, and the small, filled circles represent the protein sequences associated with each annotation. Sequences that share homology are colored with shades of the same hue. d–f) The small, filled circles and lines represent the same sequences and best hits from the previous steps.

Our pipeline follows a similar overall approach to other graph-based methods of orthology inference. First, protein sequences from annotated genomes are collected (Fig. 1a), and homology searches are conducted between all query–target pairs of genomes (Fig. 1b). The raw output from these homology searches is processed to yield best hits between pairs of sequences (Fig. 1c). Next, the network of best hits is clustered into self-consistent orthologous groups (Fig. 1d). Since genes can have multiple associated isoforms, we then implemented a novel second clustering step where orthologous groups are grouped by their parent genes, which are represented by the 2 sets of clusters in the top and bottom, respectively (Fig. 1e). Finally, representative sequences in each orthologous group are aligned (Fig. 1f). In the following sections, we discuss each of these and other steps which were omitted for clarity in greater detail.

### Input genomes and preprocessing

All annotated genomes in the genus *Drosophila* available in April 2022 were downloaded from NCBI's RefSeq database (O'Leary et al. 2016). The assemblies annotated by the NCBI eukaryotic genome annotation pipeline have passed several quality checks and all have supporting transcript evidence, so the annotations generally have highly complete gene sets as measured by BUSCO scores (Thibaud-Nissen et al. 2013; Simão et al. 2015). The *Drosophila miranda* annotation was excluded due to its unusual karyotype (Dobzhansky 1935). Other annotations were excluded after preliminary clustering showed a deficiency in the number of orthologous groups containing those genomes, indicating that their annotations were less complete (data not shown). The *Drosophila melanogaster* annotation was downloaded from FlyBase (Gramates et al. 2022). In total, the input data consist of 33 genomes, which are listed in Supplementary Table 1.

Many genes have transcripts that differ only in their UTRs, and as a result, there are many duplicate protein sequences in the annotations. Though not strictly necessary, we removed the duplicates in our pipeline, which greatly reduced the computational burden of later steps. However, we included all isoforms with distinct sequences for each gene in the subsequent BLAST searches. This differs from many orthology inference pipelines that select a representative sequence for each gene at the onset, typically the longest in practice. We took this approach to avoid arbitrarily choosing representative sequences for genes, which may result in alignment artifacts if the sequences are derived from transcripts with different intron–exon structures. Furthermore, it also allowed us to leverage the results of the initial clustering and alignment steps to make more informed choices of representative sequences, as discussed in subsequent sections.

### Extraction of best hits from BLAST output

The protein sequences in each genome annotation were searched against each other in reciprocal pairs using BLAST, yielding a list of HSPs for each query–target pair (Camacho et al. 2009). HSPs are local alignments, meaning that they do not necessarily span the entire lengths of the query and target sequences. Consequently, the search algorithm may return multiple HSPs for each query–target pair if statistically significant regions of homology are separated by poorly conserved regions or other nonhomologous sequences, e.g. alternatively spliced exons. Though the most significant HSP is often used to represent all HSPs between a query–target pair, this approach can fail to rank the pairs by their overall significance if their alignments are broken into multiple HSPs. Furthermore, since query–target pairs were later filtered by the amount overlap between their sequences, it can also exclude pairs that pass the overlap threshold even if the most significant HSP alone does not. Thus, HSPs were merged into a single object called a hit. The best hits for each query were then taken as the highest-scoring hits that passed a minimum overlap criterion and were reciprocal between the query and target sequences.

### Clustering in orthologous groups

**Fig. 2.**
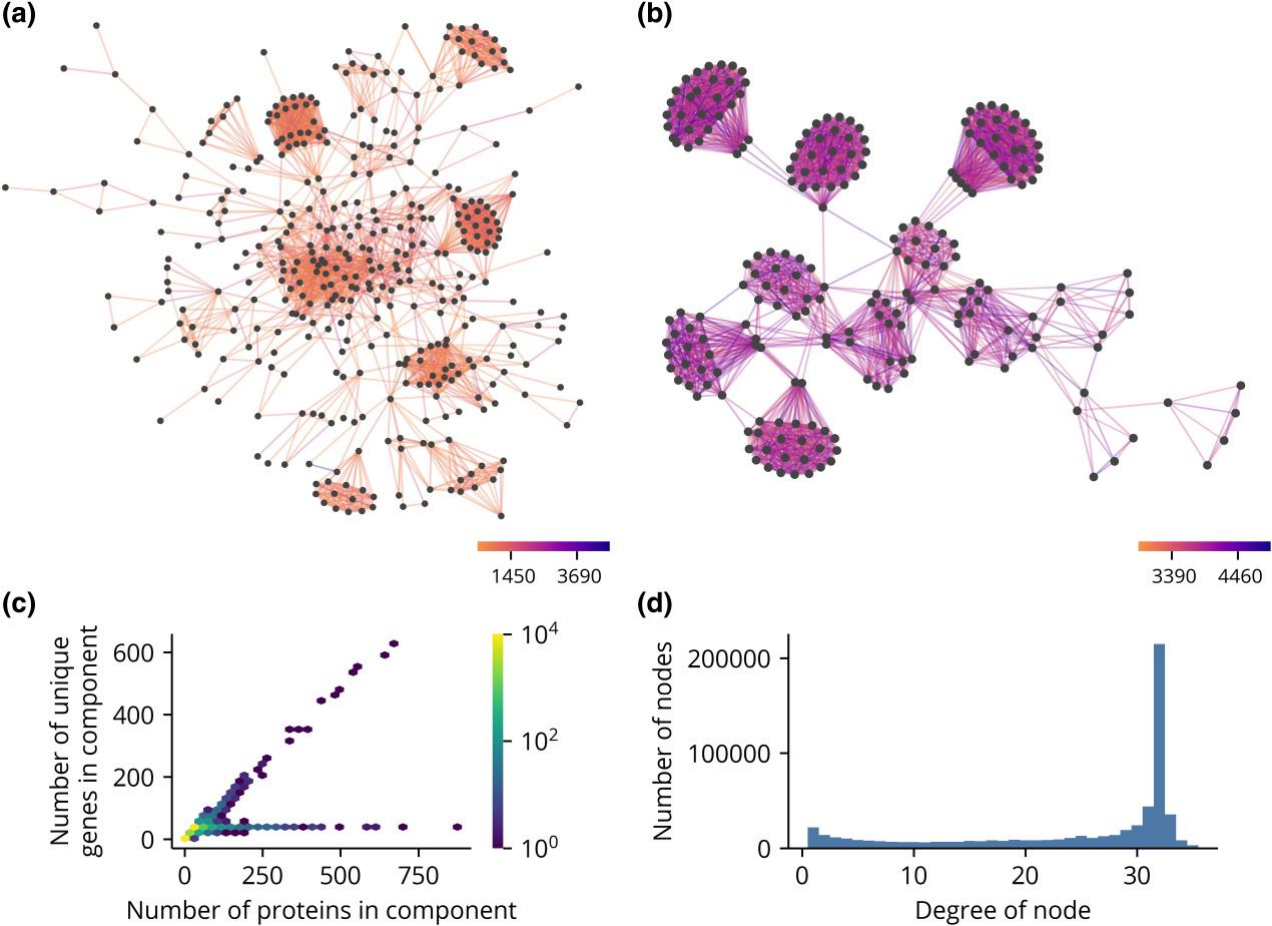
Selected connected components of hit graph and summary statistics. a, b) Two distinct connected components of the hit graph. Edges are colored by the value of their bit score. c) Hexbin plot of the number of sequences and the number of unique genes in each component. d) Histogram of number edges associated with each sequence, i.e. the degree of each node. Only the lower 99th percentile of the distribution is shown.

The best hits between sequences are naturally visualized as a graph where sequences are nodes and best hits are edges between nodes. Two connected components, sets of nodes joined by a sequence of edges, are shown (Fig. 2a, b). The sequences in the first (Fig. 2a) all contain C2H2 zinc fingers, whereas the sequences in the second (Fig. 2b) are members of the Par-1 family of serine/threonine protein kinases. In both components, some sets of nodes have a high density of edges, forming distinct clusters, whereas other nodes are only sparsely connected to their neighbors. To better understand the structure of these 2 components, we calculated the number of sequences, unique genes, and unique species in each. The first has 385, 346, and 33 sequences, genes, and species, respectively, and the second has 222, 33, and 33 sequences, genes, and species, respectively. We then plotted the relationship between the number sequences and unique genes across all components to see if this pattern holds true generally (Fig. 2c). Two distinct trendlines are apparent. The first increases linearly with the number of sequences with a slope of 1, indicating that each sequence is generally associated with a unique gene. The second is constant with an intercept of 33, indicating that the number of unique genes quickly saturates at the total number of genomes. Thus, there are generally 2 classes of components. The first is composed of many distinct genes, whereas the second is composed of many different isoforms of a single group of genes.

The diffuse networks observed in the first component class are likely the result of a combination of factors, including rapid evolution, gene duplication, and annotation errors. Regardless of their origin, these hits are not strong candidates for comparative analyses since an orthology relationship is supported by relatively few genome pairs. Instead, likely orthologs should consistently identify each other as best reciprocal hits across many genome pairs. The same is true of the hits in the second component class. Although the genes as a unit form a single orthologous group, sequences with few hits are likely non-conserved or tissue-specific isoforms. Thus, orthologous groups can be operationally defined as self-consistent clusters in the hit graph. However, sequence divergence or assembly and annotation errors may prevent a best reciprocal hit between orthologous sequences across all genome pairs. In fact, although the most common number of reciprocal hits is 32, 1 fewer than the total number of genomes, many sequences have fewer (Fig. 2d). Thus, the clustering method should require a high degree of self-consistency without demanding complete consensus.

The identification of sets of densely connected nodes in graphs is known as community detection in network analysis. While many community detection algorithms are available, only some are commonly used in the context of orthology inference. One early method that remains popular is building clusters progressively by identifying nodes that form a triangle with at least 2 other nodes in the cluster (Tatusov et al. 1997; Jensen et al. 2007). Other approaches include the MCL algorithm, which clusters graphs by simulating stochastic flow (Enright 2002; Li et al. 2003; Emms and Kelly 2015), and connected components or other single-linkage criteria (Remm et al. 2001; Linard et al. 2011; Train et al. 2017; Cosentino and Iwasaki 2018). While these methods are robust when clustering hit graphs derived from smaller or more diverse sets of genomes, they are not suitable for the large number of closely related genomes in this work since they require relatively few edges to define a cluster. For example, the MCL algorithm and connected components method assign a node to a cluster as long as it has a single edge, and triangle clustering only requires 2 edges to 2 adjacent nodes.

**Fig. 3.**
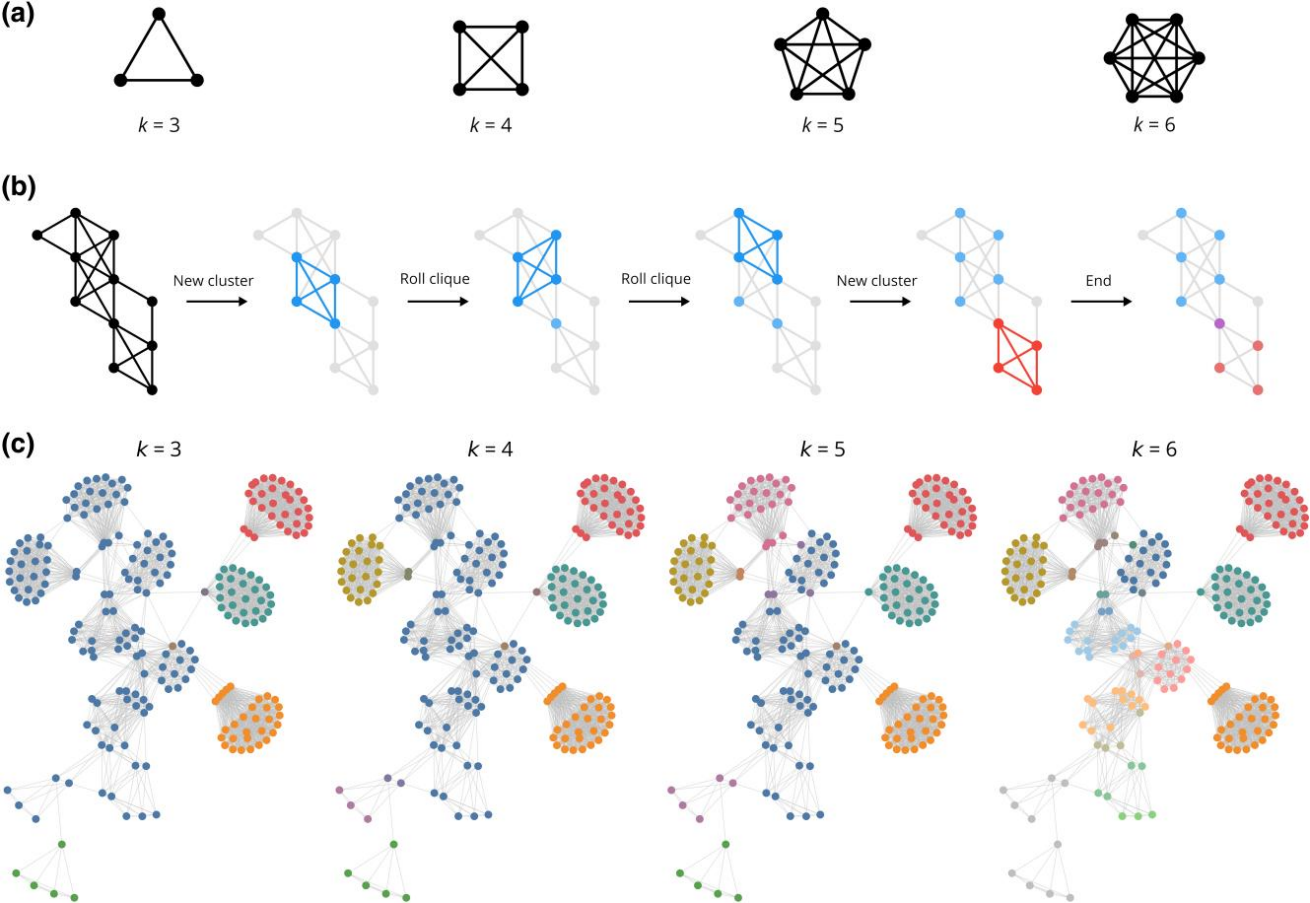
Clique percolation algorithm. a) Cliques for *k* equal to 3, 4, 5, and 6. *k* equal to 1 and 2 correspond to a single node and 2 nodes joined by an edge, respectively. b) Illustration of clique percolation algorithm where *k* = 4. c) A single component clustered by clique percolation with varying values of *k*. Nodes are colored according to their cluster. If a node belongs to multiple clusters, it uses a blend of those colors.

However, connected components and triangle clustering are special cases of the more general *k*-clique percolation algorithm where *k* equals 2 and 3, respectively. The clique percolation algorithm detects clusters by first identifying cliques, sets of nodes which are fully connected, of a specified size *k* in the graph (Fig. 3a). Clusters are then taken as the connected components of an overlap graph where an edge exists between 2 cliques if they share *k* − 1 nodes in common. An intuitive way to visualize this algorithm is by “rolling” a clique of some size *k* over the graph (Fig. 3b). More specifically, a cluster is initiated when a set of nodes which form a *k*-clique is identified. The cluster expands by shifting the *k*-clique to an adjacent *k*-clique that shares *k* − 1 nodes in common with the current *k*-clique. A cluster stops expanding when there are no adjacent *k*-cliques, and the algorithm terminates when there are no *k*-cliques which are not part of a cluster. The strength of this algorithm is its ability to exclude sparsely connected nodes from clusters with an easily tunable parameter *k*. Higher values of *k* require greater overlap between a candidate node and those already in the cluster and therefore produce tighter clusters at the cost of excluding more speculative orthology relationships (Fig. 3c). We set *k* to 4 as compromise between these concerns, yielding 22,813 orthologous groups, a plurality of which contained all 33 species (Supplementary Fig. 2).

### Addition of paralogs to orthologous groups

A weakness of the best reciprocal hits criterion is its exclusion of recently diverged paralogs. Since only the highest-scoring hits for each query are included in the graph, a paralog without a corresponding duplicate in the target genome is ignored if it is marginally more diverged than the other copy. This is corrected by adding likely paralogs to the orthologous groups. Briefly, the protein sequences in each genome were searched against themselves. If the bit score for an intra-genome hit exceeded the bit score of any inter-genome hits for the same query, the 2 sequences were identified as a paralogous pair. Orthologous groups were then supplemented with paralogs by adding the paired sequences for each of the original members of the orthologous group. Most orthologous groups contain no paralogs, and those that do generally have few relative to the original number of sequences in the group (Supplementary Fig. 3).

### Grouping orthologous groups by gene

As genes can have several annotated isoforms, each gene can be associated with several orthologous groups. However, the orthologous groups are not organized into gene-level units since they were clustered using sequence similarity only. A graph-based approach was therefore used to group orthologous groups with similar sets of parent genes. First, a gene overlap graph was constructed by defining an edge between orthologous groups if the intersection of their associated sets of parent genes is at least 50% of the smaller of the 2. Gene groups were then taken as the connected components of the resulting graph, yielding 14,909 groups. This is commensurate with the roughly 15,000 genes in each genome, which suggests that this approach has successfully clustered orthologous groups derived from a common set of parent genes.

### Initial alignment and selection of representative sequences

Since the NCBI annotation pipeline incorporates transcriptome data from a variety of sources, its inputs are heterogeneous in sequencing depth, developmental stage, and tissue of origin across different genomes. As a result, some genomes are annotated with different or multiple splice isoforms of a given orthologous gene, which can create complex networks in the resulting hit graph. For example, if the genomes are variably annotated with 1 or both of 2 distinct isoforms, the resulting graph may contain 2 clusters connected by a “bridge” formed by the genomes which contain only 1 of the isoforms. If the nodes bridging the 2 clusters form cliques with themselves and the clusters, the clique percolation algorithm will merge all the nodes into a single orthologous group where some genes have multiple associated sequences. However, these additional sequences can complicate downstream comparative analyses that may not easily generalize to genes with multiple associated sequences. Thus, in our pipeline, a single representative was chosen for each gene using an alignment-based strategy detailed in the Materials and methods section. Briefly, a statistical profile was created from an alignment of the sequences in each orthologous group, and the representative for each gene was chosen as the sequence which best matched this profile.

### Selection of single copy orthologous groups

The criteria for selecting orthologous groups for further analyses depend on the biological question under investigation. For example, studies of gene duplication will focus on orthologous groups with paralogs in some lineages but not in others. In contrast, analyses which assume functional conservation should restrict the orthologous groups to single copy orthologs since paralogs more frequently undergo functional divergence (Altenhoff et al. 2012; Pegueroles et al. 2013; Soria et al. 2014). A simple method for identifying such groups is requiring each species to have exactly 1 associated gene. However, since the probability of at least 1 missing gene annotation approaches 1 as the total number of genomes increases, this is too restrictive and fails to leverage the redundancy of closely related genomes. Instead, a set of phylogenetic diversity criteria detailed in Supplementary Table 2 were applied to ensure that the major lineages were represented in downstream analyses. Briefly, these criteria group species into sets with the requirement that a minimum number of species in each set are present. Furthermore, genome-wide analyses should select 1 orthologous group per each of the previously identified gene groups as to not bias the results toward genes with many distinct groups of isoforms. In summary, orthologous groups failing the phylogenetic diversity criteria were first removed, and the representative for each gene group was chosen as the highest-scoring orthologous group when ranked by the number species and the sum of the bit scores associated with each edge. This significantly reduced the number of orthologous groups from 22,813 to 8,566.

### Alignment refinement

**Fig. 4.**
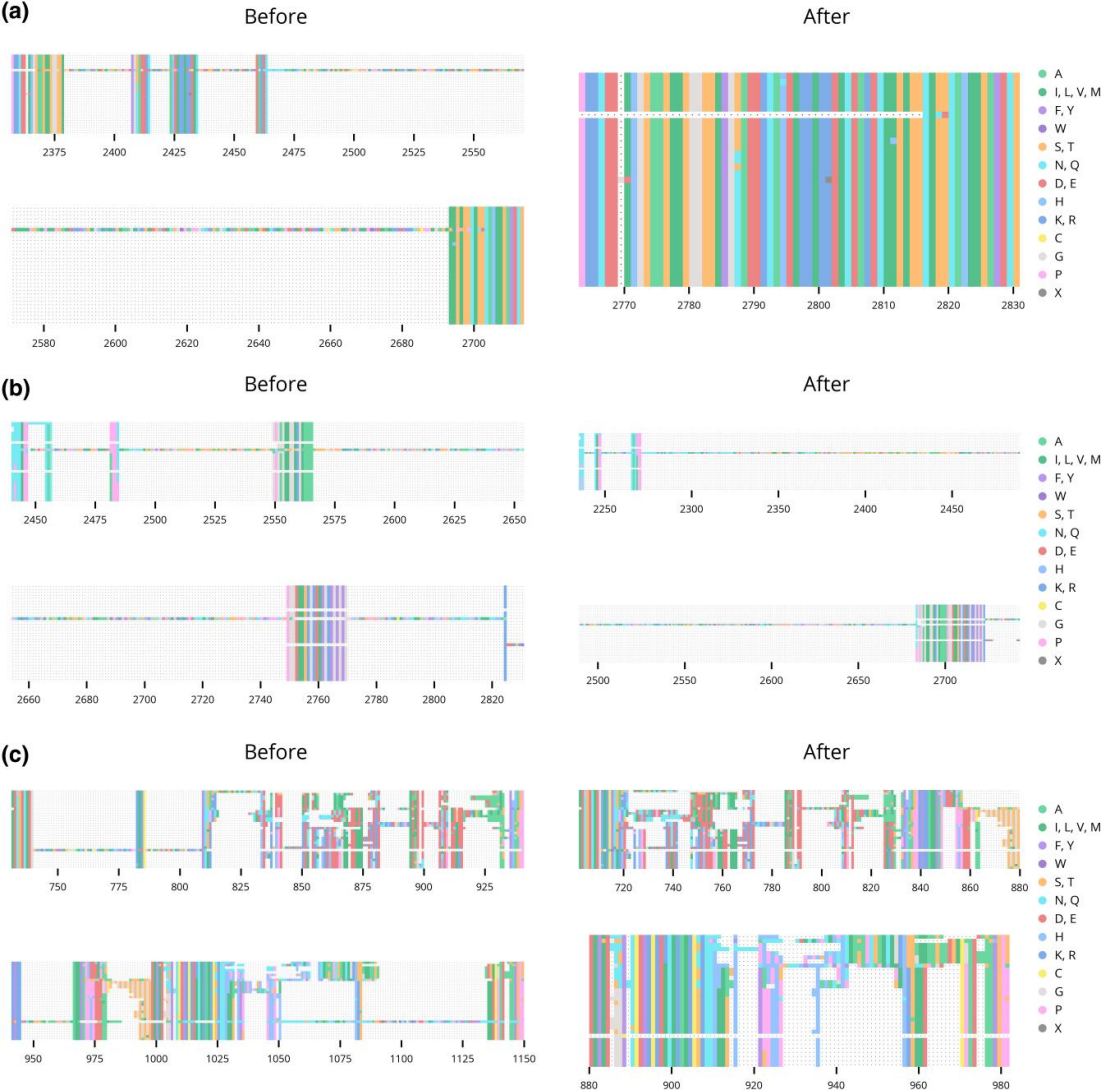
Alignment with long poorly supported segments. The alignments of representative sequences in orthologous groups 0167 a), 2770 b), and 23D9 c) before and after refinement.

Though the pipeline's quality control measures ensure a high degree of overall sequence identity between members of an orthologous group, some sequences contain long “poorly supported” segments which have no homology to most or any other sequences in the alignment. Since most common multiple sequence alignment algorithms assume that the sequences are largely homologous, these segments are sometimes “over-aligned” by forcing them into alignment where chance sequence similarities occur. Typically, these segments remain contiguous, so the alignments alternate between short runs of columns with few or no gaps and large gap-rich regions (Fig. 4a, b, left). More rarely, when long poorly supported segments are adjacent to a long gap in the same sequence, the 2 are interlaced, yielding long gaps interrupted by short segments of spurious alignment (Fig. 4c, left).

The aligner MAFFT has a mode for addressing over-alignment with a parameter, *a*_max_, that adjusts the strength of the correction (Katoh and Standley 2013, 2016). *a*_max_ varies between 0 and 1, with higher values yielding a stronger correction. While values above 0.8 completely eliminate over-alignment and successfully align highly conserved regions, the alignment as a whole is severely degraded, as even homologous sequences with a small amount of divergence are separated by gaps. Thus, in our pipeline orthologous groups were aligned in 2 stages. In the first, the sequences were aligned with a strong correction of 0.7. Highly conserved regions were identified, which divided the alignment into a complementary set of diverged regions. The sequences in each of these regions were extracted and aligned separately with a more conservative value for *a*_max_ of 0.4. The resulting “sub-alignments” were “stitched” back into their positions in the original alignment. By defining highly conserved “anchor” regions, this approach largely prevents the alignment of chance sequence similarities in long poorly supported segments (Fig. 4, right).

### Alignment curation

Although the refinement process corrects most cases of over-alignment, the alignment may still contain regions whose aligned segments have poor or inconsistent support. For example, long poorly supported segments in internal regions were not removed from the alignment since they are bounded by at least 1 consensus column to the left and right. Additionally, some regions have a significant fraction of sequences with strongly supported segments, but the observed gap pattern is discordant with the expected phylogenetic relationships. Since they are present in so few sequences, the former segments are likely artifactual, resulting from errors during assembly or annotation (biological explanations such as alternative splice sites, frameshift mutations, or transposition events are also possible, however). In contrast, the high sequence identity and clear boundaries of the segments in the latter regions suggest that they are conserved but skipped exons. Given the heterogeneous sourcing of the transcript evidence, these sequences containing these segments are likely splice isoforms specific to certain tissues or developmental stage.

Since the segments in these regions are likely the result of incorrect or incomplete annotations rather than meaningful biological variation, maintaining them in the alignments would propagate spurious homologies to subsequent analyses. This is a common issue in alignments generated by automated pipelines, so downstream analyses often focus on the strongly supported regions by removing or “trimming” columns below some threshold number of gaps or sequence identity (Castresana 2000; Capella-Gutierrez et al. 2009). This approach, however, is inadequate if the taxonomic sampling is dense, as a single indel event along a lineage containing many species can increase the number of gaps above the threshold. Moreover, as this method does not incorporate any spatial information, it can rapidly alternate between trimming and preserving columns. Thus, it can severely disrupt any analyses which are sensitive to the spatial organization of an alignment.

**Fig. 5.**
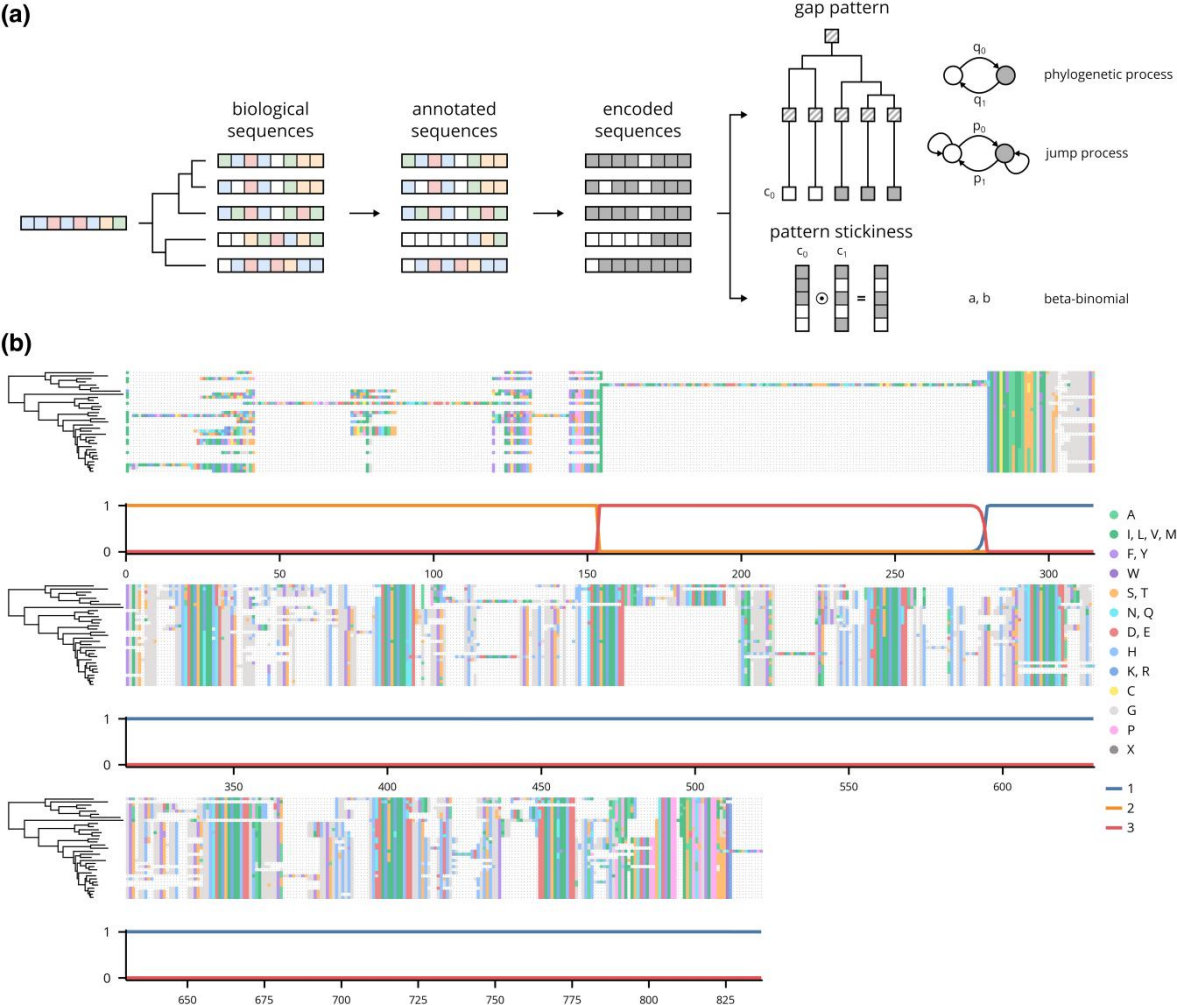
HMM emission architecture and a decoded alignment. a) Schematic of theoretical alignment generating process and corresponding probabilistic components in HMM. White and colored boxes indicate gap and non-gap symbols in the biological sequences, respectively. White and gray boxes indicate gap and non-gap symbols in the encoded sequences, respectively. c_0_ and c_1_ indicate the first and second columns in the alignment, respectively. The parameters associated with each component are shown to the right. b) The alignment of the representative sequences in orthologous group 2252 decoded using the trained HMM.

Phylogenetic hidden Markov models (phylo-HMMs) are statistical models that incorporate phylogenetic and spatial information to calculate the probability that each observation in a sequence was generated by 1 of several hidden states (Felsenstein and Churchill 1996; Siepel and Haussler 2004). Since they can evaluate both the probability of a gap pattern in a column given the known phylogenetic relationships and the local context, a phylo-HMM was used to segment the alignment into contiguous regions with different patterns of gaps. A fully specified phylo-HMM requires a fixed number of hidden states and a probability distribution for each. Thus, we identified 3 distinct types of regions in the alignments, roughly corresponding to regions with strong phylogenetic evidence, regions with a stable gap pattern discordant with the expected phylogenetic relationships, and regions with poorly supported segments. For simplicity, however, we refer to the states that generate each type of region as 1, 2, and 3, respectively. To model probability distributions for each state, we first conceptualized the observed alignments as the superposition of 2 distinct processes (Fig. 5a, left). The first is a phylogenetic process which evolves and splits a single ancestral sequence over time according to a tree. The second is the annotation process which can erroneously exclude or include segments from a sequence. The result is an alignment of annotated sequences which contains evolutionary information obscured by noise from the annotation process, shown here by the exclusion of 3 N-terminal residues in the fourth annotated sequence. To simplify modeling this behavior with an HMM, we coded the sequences into binary symbols. The distributions for each state then consisted of 2 components derived from the encoded sequences (Fig. 5a, right). The first component models the gap pattern with a Markov process. This Markov process is in turn composed of 2 subprocesses where the first is a phylogenetic process, and the second is a jump process. These subprocesses roughly correspond to changes caused by evolution and annotation, respectively. Because this first component did not fully capture the propensity for the gap patterns to remain constant, we included a second component that models the “gap stickiness” as a beta-binomial random variable by counting the number of symbols that remain constant between columns. Each component is associated with a set of parameters, and the unique parameters for each state yield its characteristic gap pattern and gap stickiness.

After the model was trained on manually labeled examples, it was used to assign a label to the columns in each alignment. Columns assigned to state 1 are the regions of interest for downstream analyses since the gaps generally follow the expected pattern given the phylogenetic tree. In contrast, columns assigned to states 2 and 3 largely corresponded to the long poorly supported segments and phylogenetically discordant regions discussed previously and were therefore removed from the alignments. In the example decoded alignment shown, the decoded states closely follow the expected patterns (Fig. 5b). Overall, 29% of alignments were trimmed of at least 1 segment or region. However, 87% of the trims were segment trims, meaning that the removed segments were largely inferred as state 3 and therefore were likely long poorly supported segments aligned to few if any other sequences (Supplementary Fig. 5).

Though the phylo-HMM removed phylogenetically incongruent insertions, some sequences still contained extensive segments of uninterrupted gaps. These segments are easily identified in regions which are otherwise highly conserved, so they are also likely the result of incorrect or incomplete annotations. However, they can also span more diverged regions, which complicates a simple rule- or threshold-based approach for identifying them. Thus, another phylo-HMM was trained to label each position in a sequence as generated by either a “missing” or “not missing” state. In this case, though, the aligned sequences were processed individually and not as aligned columns. As the previous phylo-HMM already ensured that each column has sufficient support, these labels can instead be used to exclude sequences from downstream analyses depending on the amount of tolerated overlap with the regions of interest. Overall, 15% of alignments have at least 1 sequence with a segment of “missing” data (Supplementary Fig. 7).

### Inference of species trees

**Fig. 6.**
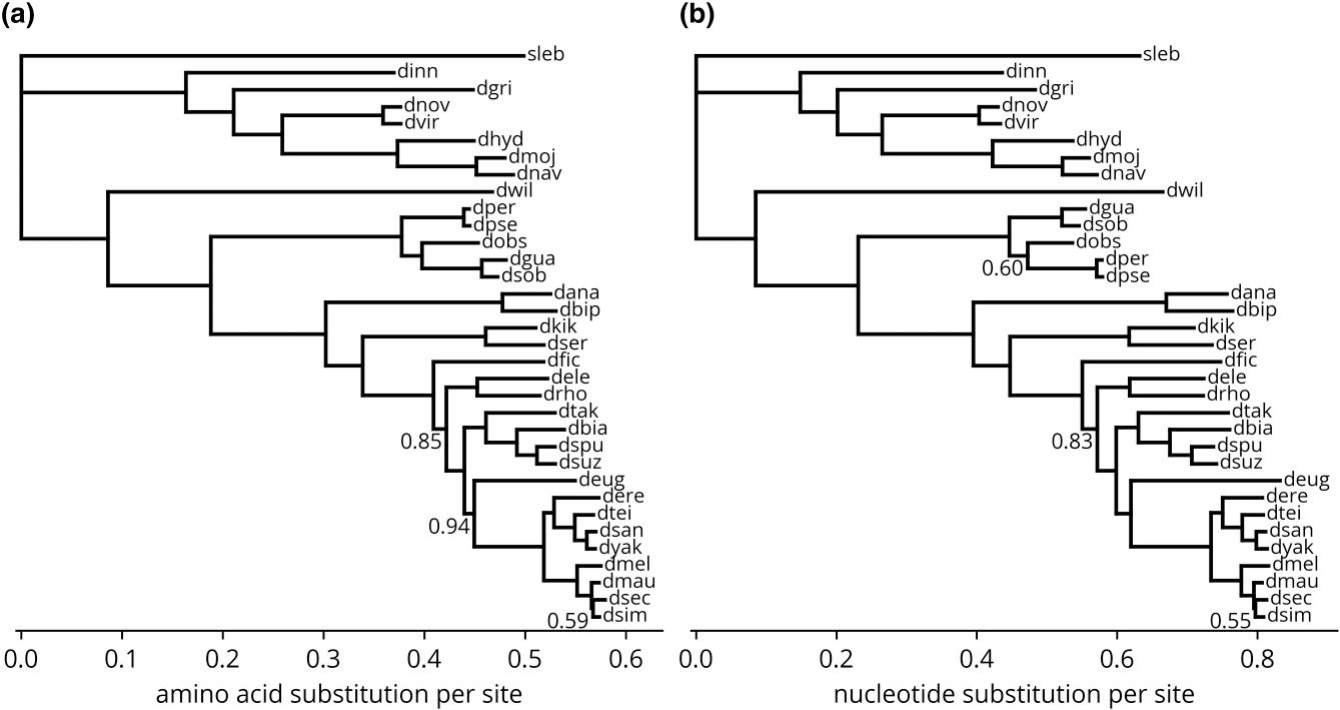
Phylogenetic tree of species. a) Consensus tree from LG model fit to meta-alignments directly sampled from the original protein alignments. b) Consensus tree from GTR model fit to meta-alignments sampled from “reverse translated” nucleotide alignments. Values at nodes are bootstrap percentages.

Many phylogenetic methods require a species tree to inform the evolutionary relationships between sequences. In fact, the phylo-HMMs discussed previously used a species tree as an input, though we omitted this detail for clarity of exposition. Therefore, to support the curation step and other downstream analyses, we sought to infer phylogenetic trees from the aligned sequences. However, since the roots of phylogenetic trees are not identifiable with commonly used time-reversible substitution models, we repeated the orthology inference pipeline with the outgroup species *S. lebanonensis*. Afterwards, we inferred phylogenetic trees using the LG model of amino acid substitution from 100 meta-alignments sampled from alignments of single copy orthologous groups. We then combined them into a single consensus tree (Fig. 6a). To provide a similar tree for the analysis of noncoding regions, we inferred phylogenetic trees using the GTR model of nucleotide substitution from 100 meta-alignments sampled from nucleotide alignments which were “reverse translated” from the protein alignments and their corresponding coding sequences (Fig. 6b).

Excluding the placement of *Drosophila obscura* (dobs), both trees have an identical topology, which is consistent with other published phylogenies (Consortium 2007; da Lage et al. 2007). Additionally, 3 other nodes have short internal branches with bootstrap percentages below 100. These results indicate that not all sites and inference methods support the same topology, potentially as a result of incomplete lineage sorting. In such cases, gene trees can differ from the species tree, and we caution against using the species tree in gene-level analyses without further investigation if deviations from the true gene trees can significantly impact the results. However, as the internal branches are generally long and the bootstrap percentages for nearly all nodes are 100, the tree is generally highly resolved and therefore appropriate for use in population- or genome-level analyses without further incorporation of uncertainty.

## Discussion

### Developments in genome assembly and annotation challenge existing methods of orthology inference

The orthologous groups and alignments yielded by this pipeline are a valuable resource for comparative studies of gene birth/death processes and protein evolution at the level of both entire proteomes and specific gene families in the *Drosophila* genus. To assist these efforts, the final alignments after curation and the labels from the “missing” phylo-HMM are provided as supplemental data. Although this work focused on single copy orthologs, other studies may require different subsets of orthologous groups that demand other preprocessing and alignment strategies. Therefore, we have included the orthologous groups and the initial alignments with and without nonrepresentative sequences in the supplemental data as well. While we anticipate these resources will remain relevant in the near term, the trends that permitted this work to substantially improve on previous efforts will render them obsolete in the coming years as more *Drosophila* genomes are assembled and annotated. However, an authoritative and lasting set of orthologous groups and alignments is not the primary goal of this work. Instead, it serves as a case study in how dense taxonomic sampling and modern genome assembly and annotation pipelines present new opportunities and challenges to the traditional techniques for identifying and aligning orthologous groups.

For example, despite many additional preprocessing steps and other tweaks introduced by later authors, the basic framework of orthology inference by clustering the hit graph has remained largely unchanged in the past 20 years (Tatusov et al. 1997; Remm et al. 2001; Li et al. 2003; Jensen et al. 2007; Linard et al. 2011; Emms and Kelly 2015; Train et al. 2017; Cosentino and Iwasaki 2018). This longevity is a testament to the robustness of the underlying idea that orthologous proteins should consistently identify each other as the most similar pairs between their genomes. Even as the number of genomes and their taxonomic density has increased dramatically, many orthology inference pipelines continue to use algorithms which were originally applied to sets of far fewer and more distantly related genomes. This mismatch in scale increases the chance of propagating annotation errors since only a small number of edges are needed to create or merge clusters. Thus, we instead applied a generalization of the triangle and connected components clustering methods called *k*-clique percolation where *k* is a tunable parameter that influences the tightness of a cluster. The optimal value of *k* for a given set of genomes is unclear and likely depends on the desired tradeoff between sensitivity and specificity. Furthermore, *k* is not necessarily a global parameter and can instead depend on the properties of each connected component. For example, 1 possibility is to take an entire component as an orthologous group if its number of unique genes and unique species are equal since all the sequences are isoforms of a single set of genes. This would effectively set equal to 1 for this component. Another approach is to make *k* a decreasing function of the density of edges, so sparser graphs are clustered more permissively. However, percolation theory or simulations may yield additional insights.

Another challenge is the annotation of multiple isoforms for a single gene. Though prior pipelines have generally selected the longest isoform as the representative before conducting the orthology searches, if the sequences do not share a common intron–exon structure, this approach can introduce artifacts or other issues during alignment. Instead, as protein sequences are increasingly derived from or linked to genomic sequences, we sought to incorporate the full annotations into the orthology inference pipeline. This, however, created 2 additional complications. First, a single gene could have several associated orthologous groups if its isoforms belonged to different clusters. Second, a single orthologous group could have several isoforms of a single gene if its isoforms were clustered together. In both cases, the presence of multiple sequences for a single gene creates ambiguities over which is the “primary” isoform. Since the first occurs at the level of orthologous groups, the orthologous groups were first grouped by the similarity of their parent genes using a graph-based strategy. Afterwards, a single representative is easily chosen as the group with the largest number of distinct species, though other criteria are possible. Since the second occurs within an orthologous group, the sequences were first aligned, and a representative for each gene was chosen as the sequence which was most concordant with this initial alignment.

The second major innovation in this work is its method for the refinement and trimming of alignments. Since sequences produced from automated annotation pipelines can contain long segments which are not homologous to most or any other sequences in their respective orthologous groups, their alignments may contain over-aligned or poorly supported regions which can introduce artifacts into downstream analyses. Thus, in refinement over-alignment is avoided by aligning the sequences in 2 stages. In the first, highly conserved regions are aligned using a strong correction for over-alignment, and in the second, more diverged regions are aligned with a weaker correction. This process usually prevents errors caused by long poorly supported segments without degrading the quality of the alignment. In trimming, a phylo-HMM is used to remove regions which are poorly supported by the phylogenetic consensus.

### Comprehensive bioinformatic analyses of proteins will depend on splice-sensitive alignments

While the combination of these 2 steps yielded high-quality alignments that are suitable for further analyses, they are an ad hoc fix for underlying issues with the gene models and alignment algorithms. The most principled solution is to optimize or supplement the gene models using the initial alignments generated by the orthology inference pipeline, which is possible with tools such as OMGene or OrthoFiller (Dunne and Kelly 2017, 2018). However, if preserving the original annotations is desired or necessary, the remaining possibility is to correct the alignments as we have done here. In fact, the errors we sought to address broadly stem from shortcomings of current alignment algorithms rather than errors in the sequences themselves. Though the scoring functions of modern multiple sequence alignment algorithms are complex, they are generally derived from models that penalize gaps with a linear or affine cost. As a result, they often interlace gaps with short, aligned segments rather than a single long gap. However, when the sequences are different isoforms of a single gene, their alignment will necessarily contain contiguous exon sized gaps. The same is true when aligning isoforms of diverged orthologs, though the relationship between their exons may be complex.

The most popular aligners for protein sequences (Clustal Omega (Sievers and Higgins 2017), MAFFT (Katoh and Standley 2013), MUSCLE (Edgar 2004), T-Coffee (Notredame et al. 2000)) do not include splice sites in their alignments, which makes them prone to aligning nonhomologous exons. Current algorithms can easily be extended to incorporate splice sites by coding them as a new symbol and preventing alignment between splice sites and amino acids, which was recently implemented in the aligner, *Mirage2* (Nord et al. 2018; Nord and Wheeler 2023). The biggest challenge in practice, however, is mapping a protein sequence to its genomic sequence to identify splice sites in the protein sequence. Although this information can in principle be derived from the GTF annotation files produced by the NCBI pipeline, annotating the splice junctions in the protein sequences themselves would facilitate splice-sensitive alignment. Though Mirage2 contains utilities which perform this mapping automatically, the NCBI annotation pipeline sometimes modifies the coding sequences of transcripts to correct for possible protein-altering mismatches or indels, labeling the resulting sequences as “low quality proteins.” The impact of these corrections on Mirage2's mapping algorithms is unclear, and as Mirage2 was released while this manuscript was in preparation, we were unable to fully investigate its performance in such cases. However, we strongly recommend that future studies aligning orthologous protein with variable exon structures explore the benefits of using Mirage2 relative to the MAFFT-based approaches described here.

These improvements would enhance rather than replace the phylo-HMM trimming method developed in this work. The model could easily be extended to include a state that outputs a splice symbol before transitioning to 1 of the states in the current architecture. This intermediate state would increase the accuracy of state inference since a splice symbol followed by a phylogenetically discordant gap pattern would strongly signal a state 2 region. The association between state 2 and skipped exons can be made explicit by requiring that transitions to and from state 2 first proceed through the splice state. This of course depends on proper labeling of the training data, which would be trivial since the boundaries between exons would be marked by splice site symbols rather than inferred from gap patterns. Unfortunately, this would not allow the phylo-HMM to label extended exon boundaries as state 2 since they would not be bounded by splice symbols to the left and right. Accordingly, the phylo-HMM would need to permit transitions between state 3 and any other state to accommodate more complex splice variants and other annotation errors. Thus, this extended phylo-HMM would combine the strengths of splice-sensitive alignment with the more heuristic approach used here. Since state 2 inferences would necessarily correspond to skipped exons, they would be suitable for analyses of this form of alternative splicing. Though state 3 inferences would not directly correspond to specific biological process, they still have value as spatially and phylogenetically aware labels for trimming poorly supported segments from alignments.

The phylo-HMM could be further enhanced by expanding its emission distribution to include more symbols in the amino acid alphabet. This would allow it to better model observed substitution patterns between specific symbols, for example the high rate of exchange between gaps and glutamine residues caused by polyglutamine tracts. The transitions between amino acids could be parametrized with a published matrix such as LG (Le and Gascuel 2008), but the transitions between amino acids and gaps would be inferred from labeled data. It is unclear if the resulting gain in accuracy would justify the increased computational burden, however.

### Accessible computational tools will facilitate future comparative studies

Though benchmarks are available for optimizing and comparing methods of orthology inference, the metrics are calculated over a set of reference genomes which are sparsely sampled over a broad taxonomic range, so it is unclear if they are informative for method designed to yield robust inferences when the genomes are highly related (Nevers et al. 2022). Furthermore, the heterogeneity of genome architectures and annotations may require quality assurance methods tailored to each set of genomes. Thus, there is likely no one-size-fits-all approach to orthology inference, and with many other standalone programs available for more standard use cases (Hieranoid (Kaduk and Sonnhammer 2017), OMA standalone (Altenhoff et al. 2019), OrthoFinder (Emms and Kelly 2019), OrthoInspector (Linard et al. 2014), Orthologer (Zdobnov et al. 2020)), we have chosen not to package the code into an end-to-end pipeline. Instead, we have devoted considerable attention to organizing and documenting the code to make it accessible to a newcomer and thereby facilitate the adaptation of specific steps to similar projects as needed.

In contrast, though many HMM packages are available for the Python programming language, we found that none were satisfactorily documented or contained tutorials to introduce HMMs and their APIs to a wide audience. We therefore refactored this code into a package available on PyPI and GitHub called Homomorph. The package itself only implements standard HMM algorithms, but the GitHub repository includes tutorials that introduce the API and implement training routines. Similar tutorials for machine-learning libraries such as TensorFlow have undoubtedly fueled the application of neural networks across diverse fields, but HMMs are also powerful models that can be more appropriate when the data obey certain statistical or structural constraints. Thus, we hope this package and its accompanying tutorials will serve as an on-ramp to HMMs and spur their greater adoption by nonspecialists.

Though databases of orthologous groups such as COGs (Galperin et al. 2020), Ensembl Compara (Herrero et al. 2016), EggNOG (Huerta-Cepas et al. 2018), OMA (Altenhoff et al. 2020), OrthoDB (Zdobnov et al. 2020), OrthoInspector (Nevers et al. 2018), and OrthoMCL (Chen 2006) will continue to be useful for comparative studies across broad taxonomic ranges, the increasing speed at which high-quality genome assemblies and annotations are produced means that no single database can encompass the most complete data. Furthermore, since many early comparative genomics studies spanned diverse branches of the tree of life, future research will likely prioritize taxonomic depth over breadth. Thus, custom sets of orthologous groups will grow more and not less common. Despite the challenges these developments pose, they also present new opportunities to bridge the gap between mutational and macroevolutionary processes.

## Data Availability

The code used to produce the results and analyses is available at https://github.com/marcsingleton/orthology_inference2023. HMM algorithms were implemented in the standalone package Homomorph which is available at https://github.com/marcsingleton/homomorph and on the Python Package Index (PyPI). The following Python libraries were used: matplotlib (Hunter 2007), NumPy (Harris et al. 2020), pandas (McKinney 2010), and SciPy (Virtanen et al. 2020). There are no primary data associated with this manuscript. All primary data are available from publicly accessible sources described in their corresponding sections. Relevant outputs are available in the supplemental files at figshare: https://doi.org/10.25387/g3.23787168. File S1 contains the orthologous groups generated by 4-clique percolation with paralogs added. File S2 contains the initial alignments of all sequences in each orthologous group. File S3 contains the refined alignments of the representative sequences in each single copy orthologous group. File S4 contains the insertion phylo-HMM model parameters and the alignments of the single copy ortholog groups after trimming. File S5 contains the missing data phylo-HMM model parameters and the missing segments identified for each sequence in each single copy orthologous group. Missing segments are given as Python slices in the following form: start0-stop0, start1-stop1,…,startn-stopn. If the slices field is empty, then no missing segments were identified in that sequence. File S6 contains the consensus phylogenetic trees fit using the LG, GTR, and GTR2 substitution models.
